# Effects of Gelam and Acacia honey acute administration on some biochemical parameters of *Sprague Dawley* rats

**DOI:** 10.1186/1472-6882-14-146

**Published:** 2014-05-04

**Authors:** Suhana Samat, Nor Azmi Md Nor, Fuzina Nor Hussein, Wan Iryani Wan Ismail

**Affiliations:** 1 Clinical BioPharmaceutics Research Group (CBRG), Brain and Neuroscience Core, Universiti Teknologi MARA, 40450, Shah Alam, Selangor Darul Ehsan, Malaysia and Faculty of Pharmacy, Universiti Teknologi MARA, Puncak Alam Campus, 42300 Bandar Puncak Alam, Selangor Darul Ehsan, Malaysia; 2 Faculty of Veterinary Medicine, Universiti Putra Malaysia, 43400 Serdang, Selangor Darul Ehsan, Malaysia

**Keywords:** Acacia honey, Acute consumption, Gelam honey, *Sprague Dawley* rats

## Abstract

**Background:**

Since ancient times, honey has been used for medicinal purposes in many cultures; it is one of the oldest and most enduring substances used in wound management. Scientific evidence for its efficacy is widely studied, but systemic safety studies are still lacking. It is essential to study the impact of consumption of honey on the health and proper development of the consumer. Therefore, the present study was designed to observe the effects of acute administration (14 days) of Gelam honey (GH), a wild harvesting honey and Acacia honey (AH), a beekeeping honey, on male and female *Sprague Dawley* (SD) rats.

**Methods:**

An acute oral study was performed following OECD test guideline 423, with minor modifications. In the study, GH, AH and sucrose (S) were administered at 2000 mg/kg body weight. Animals were obs*e*rved for the next 14 days. Gross pathology was performed at the end of the study. Animals were observed for mortality, morbidity, body weight changes, feed and water intake. Clinical biochemistry, gross pathology, relative organ weight and histopathological examination were performed.

**Results:**

Rats fed with honey did not exhibit any abnormal signs or deaths. Results showed a decrease in weight gain and energy efficiency, but significantly increased in total food intake and total calories in female rats fed with GH, compared to control (p < 0.05). Nevertheless, a significant increase in body weight was observed in male rats in all honey-treated groups. Male rats fed with AH significantly decreased in total food intake, total calories and energy efficiency. Both male and female rats fed with GH displayed a significant decrease in triglycerides compared to control group. Hepatic and renal function levels were within acceptable range. The gross necropsy analysis did not reveal changes in any of the organs examined.

**Conclusions:**

Our results suggest that acute consumption of GH and AH at 2000 mg/kg body weight of male and female SD rats has some discrepancy effects on biochemical parameters but in line with OECD regulation. Gelam honey may have potential in controlling weight gain and triglyceride levels in female rats compared to Acacia honey. SD rats have some effect on biochemical parameters, an exploration of which would make for intriguing analysis.

## Background

Honey, a sweet and viscous fluid, has medicinal properties at both preventive and curative levels. Since ancient times it has been known to have anti-bacterial, antioxidant and wound-healing constituents [1]. Moreover, honey also exhibits anti-tumor activity, with pronounced anti-metastatic and anti-angiogenic effects [2], and anti-bacterial, anti-inflammatory, immune-stimulant, anti-ulcer and wound-/burn-healing properties [3]. Various signalling pathways mediated by honey and its major components (such as chrysin, pinobanksin, vitamin C, catalase, and pinocembrin) have been unravelled recently, including stimulation of tumor necrosis factor-alpha (TNF-α), inhibition-cell proliferation, apoptosis induction and cell cycle arrest, as well as lipoprotein oxidation [4]. Apart from the components mentioned above, honey also contains a variety of other biologically active compounds, such as flavonoids, vitamins, and antioxidants, as well as hydrogen peroxide (H_2_O_2_), which make it an extraordinary food, possessing potent and varied medical properties [5].

Honey is produced from many different floral sources, and its biochemical and pharmacological activities vary depending on its origin and processing. In Malaysia, varieties of honey may be divided into floral honey (such as honey harvested from trees including Gelam, Tualang, Pineapple, and Coconut) and honeydew honey (for instance Acacia honey) [6]. For this study, a floral honey (Gelam honey, or GH) and a honey from honeydew honey (Acacia honey, or AH) were selected. They are both widely produced and consumed in Malaysia, and both exhibit antioxidant activity [7]. GH is produced by *Apis mellifera*, a honey bee, from *Melalucae cajupati* tress, particularly from the eastern part of Malaysia and harvest widely from the forest. It contains high levels of polyphenols and of non-phenol contents, compared to pineapple and coconut honey. AH is also produced by *A. mellifera,* but from the *Acacia magnium* plants, especially in north Malaysia and collected from a beekeeping farm. It also contains phenolic acids and flavonoids [8,9].

Rigorous research studies have been conducted on honey; however, only a few studies to date have taken cognizance of the possible consumption effects of GH and AH. For instance, Kassim et al. (2012) [1] documented acute analysis of GH on mice (Balb/c mice) and New Zealand white rabbits at doses of 10, 60, 300, and 600 mg/kg, diluted with saline. However, an acute study of GH and AH with doses of 2000 mg/kg body weight on *Sprague Dawley* rats according to OECD guideline has not been carried out, even though these rats have been used intensively in honey studies. Therefore, this study aims to observe the effects of acute administrations of GH and AH at single doses of 2000 mg/kg of body weight.

## Methods

### Sample collection

Gelam honey (GH) was collected from Gelam forest, Terengganu, Malaysia. Acacia honey (AH) was purchased from beekeeping farm under the Department of Agriculture, Johor, Malaysia. The samples were irradiated with 25 kGy gamma radiation using the radioactive source cobalt 60 (model JS8900), at the Malaysian Nuclear Agency (MINT), Selangor, Malaysia [10]. The irradiated honey was then kept at 4°C, away from direct sunlight, in amber bottles. Sucrose (S) was purchased from Sigma-Aldrich St. Louis, MO, USA.

### Experimental animal husbandry

The experimental protocol was approved by the Research Committee on the Ethical Use of Animals (UITM Care), Reference No. 05/2012. Twenty healthy *Sprague Dawley* (SD) rats of both sexes were obtained from Laboratory Facilities of Animal Management (LAFAM), University Technology MARA (UITM), Puncak Alam, Selangor, Malaysia. At the commencement of study, each rat was eight weeks old and weighed between 180 and 220 g. The rats were housed at one rat per cage and maintained in standard environmental conditions under an ambient temperature of 25 ± 2°C and 40-65% relative humidity, with a 12-hour light/dark cycle. They were fed with certified rodent food (Rodent Diet Speciality Feeds, Glen Forrest, Australia) and drinking water was available ad libitum. The animals were acclimatized for five days prior to the commencement of the study, and appropriately labelled. A completely randomised design was used to divide the rats into four groups, each made up of five females and one male. The control rats were orally administered with distilled water and rodent food ad libitum (Group 1), fed with GH (2000 mg/kg body weight) (Group 2), fed with 2000 mg/kg body weight of AH (Group 3), and fed with sucrose (2000 mg/ kg body weight) (Group 4).

### Acute administration test

In the study, the acute oral test was performed according to the guidelines of the Organisation for Economic Co-operation and Development (OECD) for the testing of chemicals, TG 423 with slight modifications [11]. It measures adverse effects following oral administration of a single dose of a substance (or multiple doses given within 24 hours) such as general behaviour, respiratory pattern, cardiovascular signs, motor activities, reflexes, and changes in skin and fur texture. From this experiment, the LD_50_ (or median lethal oral dose, which is the statistically derived single dose of substance that can be expected to cause death in 50% of animals when administered by the oral route) was determined. The LD_50_ value is expressed in terms of weight of test substance per unit weight of test animal (mg/kg) [11].

Initially, rats were deprived of food, except for water, for twelve hours, and weighed prior to the experiment. Test substances at a dose of 2000 mg/kg body weight were given orally, once to each group, but the control group was administered with distilled water. The test groups (Groups 2, 3 and 4) received single oral doses of 2000 mg/kg body weight of GH, AH and S respectively. The doses given were calculated according to each animal’s body weight at the week of treatment specified (Table 1). Body weights of the rats were then recorded every day. Meanwhile, mortality and the clinical signs of toxicity were observed at 0.5, 1, 2 and 4 hours, and thereafter once a day for the next 14 days. On day 15, the overnight-fasted animals (water allowed) were euthanized using diethyl ether and subjected to gross pathological examination of all the major internal organs, such as heart, lung, liver, kidney and spleen.

**Table 1:** Body weight of rats in the acute toxicity study of the treated groups – Control, Gelam honey (GH), Acacia honey (AH) and Sucrose (S)

**Group**	**Male**		**Female**	
	**Initial body weight**	**Final body weight**	**Initial body weight**	**Final body weight**
Control	187.14 ± 8.89	272.68 ± 9.43	210.14 ± 6.70	279.70 ± 8.66
(GH)	187.32 ± 9.34	275.22 ± 10.75	204.98 ± 2.71	273.04 ± 9.93
(AH)	184.52 ± 4.52	279.58 ± 6.26	206.14 ± 2.87	272.72 ± 12.23
(S)	187.46 ± 10.46	279.94 ± 11.06	208.62 ± 5.40	286.90 ± 9.19

Results are reported as mean ± SEM (n = 5).

### Body weight and meal pattern analysis

The body weight (BW) of each rat was recorded once per week, and the differences in BW were noted. In the meal pattern analysis, the amount of food and water consumed was measured weekly by subtracting from the quantity of food and water supplied initially. Food efficiency was calculated once, at the end of the study. The total number of kilocalories that each rat consumed was determined by multiplying the caloric content of 1 gram of each diet by the total quantity of food eaten [12].

### Biochemical analysis

Rats were fasted overnight, anaesthetised using diethyl ether, and sacrificed at the end of the experiment. Blood samples were freshly collected through cardiac puncture, and the samples were stored in EDTA tubes for serum biochemical assay. The blood samples were centrifuged at 2 000 revolutions per minute (rpm) for 15 minutes at 4°C. The clear serum obtained was separated and labelled for analysis of serum levels for the hepatic function tests, including aspartate aminotransferase (AST), alanine transaminase (ALT), a renal function test (urea and creatinine) and serum lipid profile (glucose, triglycerides (TG) and total cholesterol) [13]. These levels were determined using an Auto Analyser (ILAB 300 Plus Clinical Chemistry Analyser, Milano, Italy) according to manufacturer’s protocols.

### Histological evaluation

A comprehensive gross observation was carried out on the internal organs, such as the liver, spleen, lung, kidneys and heart. They were observed for any signs of abnormality and for the presence of lesions owing to any effects of the administration of GH, AH and S [13]. The organs were then carefully dissected, cleaned of any fats and weighed. The relative organ weight (ROW) of each organ was then calculated according to the following equation:

$$\mathrm{ROW}=\left(\mathrm{absolute}\;\mathrm{organ}\;\mathrm{weight}\;\left(g\right)\times 100\right)/\mathrm{body}\;\mathrm{weight}\;\mathrm{of}\;\mathrm{rat}\;\mathrm{on}\;\mathrm{sacrifice}\;\mathrm{day}\;\left(g\right)$$

Each organ was then preserved in 10% buffered formalin for subsequent histopathological examination. The tissues were embedded in paraffin, and then sectioned; the sections were cut at 4-5 microns with the rotary microtone, stained with hematoxylin and examined microscopically [14].

### Statistical analysis

Results were expressed as mean ± standard average mean (SEM). Statistical significance was determined by one-way analysis of variance (ANOVA). Values with a confidence level of p ≤0.05 were considered significant.

## Results

### Acute administration study

There were no treatment-related deaths and no abnormal signs developed in any group throughout the 14-day study. Moreover, throughout the study, no apparent differences in physical activity or other behaviours, no significant changes in the nature of stool, urine and eye colour of any rat, no diarrhoea, salivation, convulsion, sleep or coma (which are signs associated with oral toxicity) and no significant loss of fur or skin lesions were observed. The acute study showed that rats fed with GH, AH and S did not result in any mortality; thus, we cannot determine LD_50_ from the study, i.e. the LD_50_ value would be greater than 2000 mg/kg body weight. According to the Globally Harmonised System of Classification and Labelling of chemicals, GH and AH at 2000 mg/kg body weight may be classified as Category 5, which is safe for consumption.

### Body weight

**Figure 1:**
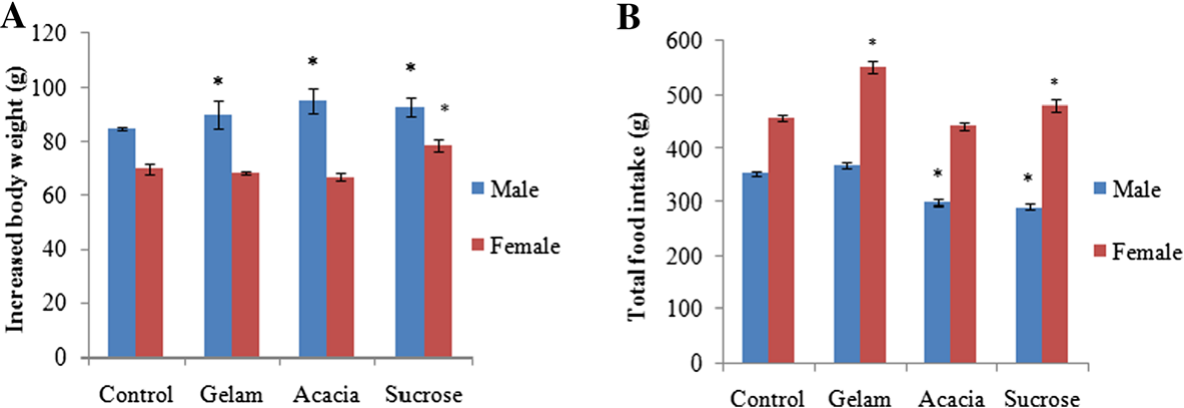
**Effects of treated dose of control, Gelam honey (GH), Acacia honey (AH) and sucrose on (A) increased body weight in grams (g), (B) Total food intake in grams (g).** Values are expressed as mean ± SEM (n=5). *Significant difference (p < 0.05) versus control.

In female rats, body weight gain of the group fed with GH and AH showed slightly reduction compared to the control group (Table 1 and Figure 1). Meanwhile, body weight gain observed in male rats in GH, AH and S groups showed a significant increase (p < 0.05) (Table 1 and Figure 1). The percentage reductions in body weight of rats fed with GH and AH were 2.38% and 2.49% respectively, compared to control.

### Meal pattern

**Figure 2:**
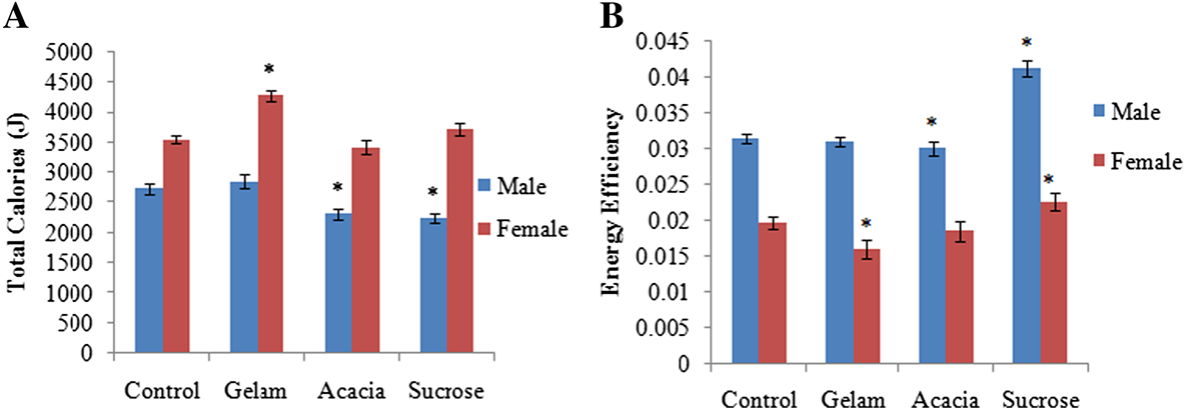
**Effects of treated dose of control, Gelam honey (GH), Acacia honey (AH) and sucrose groups on (A) total calories (J) and (B) energy efficiency.** Values are expressed as mean ± SEM (n=5). *Significant difference (p < 0.05) versus control.

Total food intake for the female rats fed with GH exhibited significant increase compared to the control group. However, mean total food intake in male rats fed with AH and S decreased significantly. Concomitantly, total calories for female rats fed with GH were significantly increased (4277.71 J) compared to the control group of rats (3541.63 J). Meanwhile, calories in male rats fed with S and AH were significantly (p < 0.05) decreased (2244.33 J) and 2308.07 J, when compared to control and GH groups (Figure 2). Male rats fed with sucrose had significantly increased energy efficiency (0.0412) compared to control (0.0314) and GH (0.031). Female rats fed with GH (0.016) and AH (0.0185) respectively demonstrated decreased in an energy efficiency compared to the control (0.0197).

### Hepatic function indices

The results obtained for serum levels of AST showed a significant decrease in rats fed with GH and with AH (Table 2), but not for rats fed with S (in male rats). However, in female rats, AH and S levels showed a significant increase (to 154 U/L and 146 U/L) compared to control (112 U/L). Meanwhile, results for ALT levels showed a significant increase for all treated groups, for both male and female rats.

**Table 2:** Biochemical changes in rats after 14 days of acute oral administration of Control,Gelam honey (GH), Acacia honey (AH) and sucrose (S)

	**Male**				**Female**			
	**Control**	**(GH)**	**(AH)**	**(S)**	**Control**	**(GH)**	**(AH)**	**(S)**
Glucose (mmol/L)	11.36 ± 0.25	17.60 ± 0.42^*^	21.84 ± 0.33^*^	22.91 ± 0.42^*^	11.80 ± 0.76	12.62 ± 1.52	12.42 ± 0.55	12.70 ± 0.67^*^
Urea (mmol/L)	4.50 ± 0.50	6.16 ± 0.38^*^	7.52 ± 0.40^*^	8.16 ± 0.60^*^	5.40 ± 0.55	5.24 ± 0.44	5.61 ± 0.37	5.82 ± 0.27^*^
Creatinine	20.98 ± 0.81	41.83 ± 2.05^*^	52.00 ± 2.74^*^	60.44 ± 0.61^*^	38.00 ± 2.50	48.00 ± 2.74^*^	54.00 ± 5.48^*^	54.00 ± 5.47^*^
Cholesterol (mmol/L)	1.46 ± 0.01	1.63 ± 0.03^*^	1.59 ± 0.10^*^	1.77 ± 0.10^*^	3.58 ± 0.11	3.48 ± 0.07	3.63 ± 0.07	3.58 ± 0.06
TG (mmol/L)	1.42 ± 0.01	0.72 ± 0.02^*^	1.95 ± 0.16^*^	1.92 ± 0.01^*^	2.48 ± 0.18	1.46 ± 0.21^*^	2.22 ± 0.11^*^	2.66 ± 0.11^*^
AST (U/L)	108.76 ± 0.55	67.24 ± 2.76^*^	72.22 ± 0.23^*^	123.94 ± 0.90^*^	112.00 ± 4.47	108.00 ± 8.36	154.00 ± 5.48^*^	146.00 ± 6.52^*^
ALT (U/L)	41.86 ± 0.52	45.28 ± 0.33^*^	47.22 ± 0.45^*^	49.36 ± 0.23^*^	40.24 ± 0.45	65.00 ± 5.70^*^	68.00 ± 4.47^*^	55.00 ± 7.07^*^

Results are reported as mean ± SEM (n = 5); ALT: alanine aminotransferase; AST: Aspartate Aminotransferase.

^*^Significant different (p < 0.05) versus control.

### Renal function parameters

A dose administration (2000 mg/kg BW) of GH, AH and S resulted in significant increased levels of urea and creatinine in male rats (Table 2). In urea and creatinine levels, GH showed significant (p < 0.05) increases (6.16 and 41.8 mmol/L respectively) compared to control (4.50 and 20.98 mmol/L respectively). Urea and creatinine levels exhibited the highest value (8.16 mmol/L and 60.44 mmol/L respectively) in the S group compared to control group (4.50 mmol/L and 20.98 mmol/L respectively) of male rats. However, in female rats, urea level in AH was not significantly increased compared to control. Meanwhile, the GH group showed no significant change (5.20 mmol/L) in urea level compared to control (5.40 mmol/L) (Table 2).

### Serum lipid profile

Serum TG concentrations were significantly (p < 0.05) decreased in both male and female rat groups fed with GH compared to control. Meanwhile, for female rats, cholesterol level tests of AH did not show a significant increase compared to control. The significantly decreased levels in the TG group indicate that treatment might not have had lipogenic effects. All cholesterol, glucose and TG level tests for rats fed with GH and AH were within range [11], except for the TG test in female rats fed S, and the glucose test in male rats fed with AH and S.

### Relative organ weight

The absolute and relative organ weights of the isolated hearts, spleens, kidneys, lungs and livers from the groups were recorded and calculated (Table 3). Gross necropsy findings did not reveal changes in any of the organs examined. The relative organ weight for kidneys recorded at the end of the study showed a significant (p < 0.05) decrease for both male and female AH rats compared with the control group. However, other organs showed no significant change in relative organ weight (ROW) in either male or female rats. As shown in Table 3, the male group fed with AH at doses of 2000 mg/kg BW showed significantly lower weights for kidney than those of the control group. Slight changes were found in the weights of other internal organs, which may be due to the variation in size of the internal organs in each rat. Internal organ weights for the GH and AH rats were not significantly changed relative to those of the control group; except for the S/liver weight, which was significantly increased (3.49 g and 3.71 g) for both male and female rats compared to control (3.11 g and 3.01 g). Gross and histopathological examinations further confirmed that the substance did not cause any tissue damage.

**Table 3:** Effects in treated groups of Control, Gelam honey (GH), Acacia honey (AH) and sucrose (S) on relative organ weights (ROW) of both male and female rats

	**Male**				**Female**			
	**Control**	**(GH)**	**(AH)**	**(S)**	**Control**	**(GH)**	**(AH)**	**(S)**
Heart	0.28 ± 0.02	0.28 ± 0.02	0.28 ± 0.01	0.28 ± 0.02	0.24 ± 0.01	0.31 ± 0.01^*^	0.28 ± 0.03	0.30 ± 0.01^*^
Spleen	0.16 ± 0.01	0.20 ± 0.01^*^	0.20 ± 0.01^*^	0.19 ± 0.02^*^	0.49 ± 0.01	0.57 ± 0.01	0.61 ± 0.04^*^	0.72 ± 0.01^*^
Kidney	0.58 ± 0.02	0.6 ± 0.01^*^	0.54 ± 0.05^*^	0.59 ± 0.03	0.55 ± 0.03	0.60 ± 0.04^*^	0.54 ± 0.03	0.64 ± 0.01^*^
Lung	0.32 ± 0.11	0.36 ± 0.04^*^	0.32 ± 0.01	0.32 ± 0.03	0.31 ± 0.01	0.32 ± 0.01	0.32 ± 0.01	0.31 ± 0.01
Liver	3.11 ± 0.11	3.10 ± 0.06	3.33±^,^0.25^*^	3.49 ± 0.18^*^	3.01 ± 0.01	3.15 ± 0.06^*^	3.49 ± 0.06^*^	3.71 ± 0.10^*^

Results are reported as mean ± SEM (n = 5); ^*^significant different (p < 0.05) versus control.

### Histopathology

**Figure 3:**
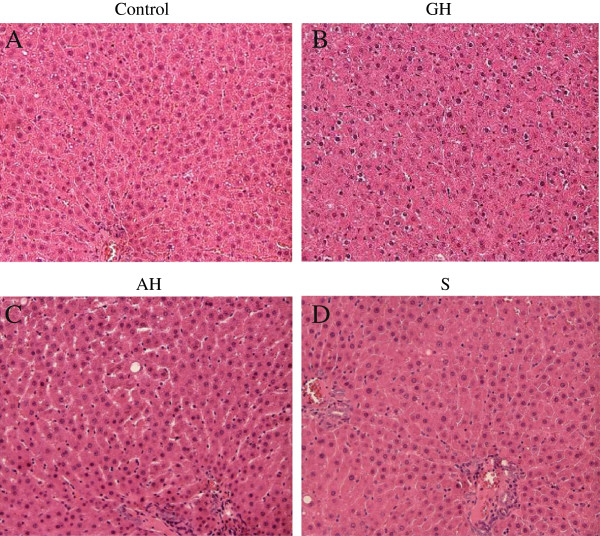
Photomicrograph of the male rat; A) control liver and male rat orally administered with (B) GH, (C) AH and (D) S at a dose of 2000mg/kg body weight for 14 days (HE staining ×200).

**Figure 4:**
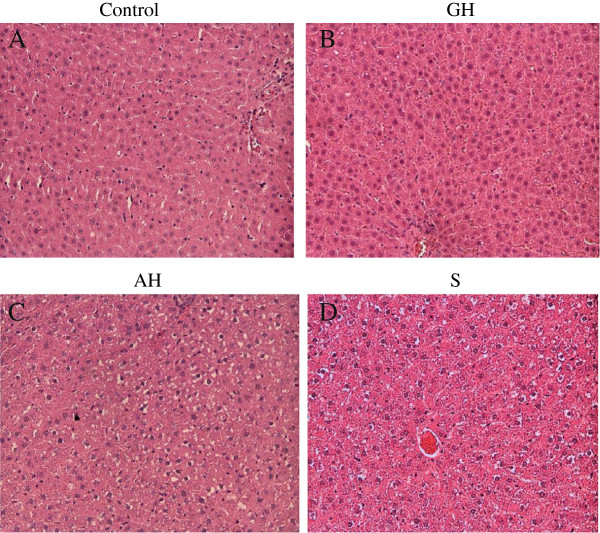
Photomicrograph of the female rat; (A) control liver of a female rat and orally administered with (B) GH, (C) AH and (D) S at a dose of 2000mg/kg body weight for 14 days (HE staining x200).

For the histological investigation, no pathological changes were observed in the livers of animals in any group (Figure 3). The macroscopic observation of the organs did not present any significant morphological or haemorrhagic changes due to the administration of any test substance. Other organs (including spleen, lung, kidneys and heart) showed no sign of pathological changes compared with the corresponding organs of the control group. For the acute test, the livers of rats on doses of 2000 mg/kg body weight (Figure 3) of GH, AH, or S did not show any changes compared to control in both male and female rats. There were no pathological changes in healthy control livers, which showed normal lobular architecture with a central vein and radiating hepatic cords [Figure 3(b)-(d)] compared to control [Figure 3(a)]. Rats fed with GH and AH alone showed no sign of damage [Figure 3(b), (c)] when compared to the control. It was composed of hexagonadal or pentagonadal lobules with central veins and peripheral hepatic triads or tetrads embedded in connective tissue. Hepatocytes were arranged in trabecules running radiantly from the central vein, and were separated by sinusoids containing Kupffer cells. They were regular and contained a large, spheroidal nucleus with a distinctly marked nucleolus distribution. Similarly, female rats fed with GH and AH showed hepatocytes in most sections [Figure 4(a)-(d)] containing clear, pale staining nuclei with one to two nucleoli [Figure 4(b)]. The cytoplasm of most hepatocytes was pale and eosinophilic, with finely granular basophilic inclusions. The hepatic sinusoids and central veins were predominantly clear of erythrocytes.

## Discussion

Honey has been used for medicinal purposes in many cultures since ancient times [4]. It is one of the oldest and the most enduring substances used in wound management [15]. Scientific evidence for its efficacy has been widely studied [15,16], but systemic safety studies are still lacking. Therefore, it is essential to study the impact of consumption of honey in animal models to ensure its impact on health compared to that of sucrose; the major sweetener used worldwide [17]. In this study, Gelam honey (GH) and Acacia honey (AH) were selected because they are extensively consumed in Malaysia [9].

In the acute study, a dose of 2000 mg/kg body weight, and male and female was selected, according to OECD test guideline 423. It is the limit test at the highest starting dose level and consistent approach in studying the low or non-toxicity nature of test material [11].

Results from the study reveal that the oral administration of doses of GH, AH and S did not cause abnormal or alter any behavioural or physiological state of the rats in the acute tests. In female rats, body weight gain and energy efficiency of the group fed with GH showed a no significant decrease compared to the control group. Meanwhile, total food intake and total calories of the rats demonstrated a significant increase. In contrast, only body weight gain of the male rats fed with AH showed a significant increase (p < 0.05) compared to control group. Weight gain by some rats could be ascribed to the nutritive compounds in honey, and its androgenic properties, since androgens exhibit anabolic activity [17]. However, hormonal factors in female rats consider playing a role in the study and required further investigation.

Analysis of blood parameters is relevant to risk evaluation of alterations in human systems [18]. In the study, no significant alterations of the biochemical parameters could be attributed to the dose given in either male or female rats. The kidney, a sensitive organ, is known to be affected by a number of factors, such as drugs or harmful substances, that ultimately lead to renal failure [19]. Assessment of possible renal damage due to GH and AH administration was made by assaying serum urea and creatinine levels. Only male rats fed with GH, AH and S resulted in significant increase levels of urea and creatinine. However, the value is still within acceptable range except for rats fed with S [11,20]. Similar finding observed on the levels of AST and ALT; which are considered to be sensitive indicators of hepatocellular damage, and can provide a quantitative evaluation of the degree of damage to the liver [21]. With the exception of sucrose, it is reasonable to deduce that GH and AH did not cause any damage to the liver and kidneys. This is further confirmed by a histological assessment of these organs, and the fact that plasma cholesterol levels remained unaffected – the latter being an indirect indicator of liver function [20].

Increment in the relative organ weight in male and female rats fed with GH, AH and S considers normal. No difference was observed between the control and the GH- and AH-fed groups in terms of relative organ weight and structure of the other organs. Therefore, the acute study indicates that neither GH nor AH ingestion induced detrimental changes or morphological alterations in these organs, and they can be classified under Category 5, with LD_50_ value greater than 2000 mg/kg body weight (Globally Harmonised System of Classification and Labelling of Chemicals, 2009). This information gives us the direct relevance of GH and AH, as the dose concentration is appropriate for both human and animal consumption. This supports the wide use of honey as a therapeutic remedy, both topically and orally, and in consumer products [21].

## Conclusion

The results from this study indicate that daily consumption for 14 days of Gelam honey or Acacia honey may have positive effects on body weight, meal pattern and biochemical parameters of consumers, depending on the concentration and the gender of rats. LD_50_ of GH and AH are both greater than 2000 mg/kg body weight, indicating the safety of the test substances. Thus, GH and AH can be classified under Category 5 of the Globally Harmonised System of Classification and Labelling of Chemicals. Present results substantiate (at least in part) the impact of consumption of GH and AH, which was found to be in line with the long history of uses of honey as remedies. However, wild harvesting honey (Gelam honey) exhibit more potential effects compared to beekeeping honey (Acacia honey) in term of controlling weight gain and cholesterol level. Therefore, future work will focus on the on biochemical analysis of the long-term impact on health of the consumption of Gelam and Acacia honey.

## Competing interests

The authors declare that they have no competing interests.

## Authors’ contributions

SS planned and carried out the experiments on Gelam honey, was involved in data interpretation, and prepared the manuscript, including revisions. NAMN planned and carried out the experiments on Acacia honey. FNH designed and planned the experiments from the animal aspect, and contributed to data interpretation. WIWI acted as project leader and advisor to this study (mainly in the use of honey in treatment), and contributed to data interpretation and troubleshooting, as well as manuscript and revision editing. All authors read and approved the manuscript.

## Pre-publication history

The pre-publication history for this paper can be accessed here:

http://www.biomedcentral.com/1472-6882/14/146/prepub
